# Supplementary material for: Rational-Based Discovery of Novel β-Carboline Derivatives as Potential Antimalarials: From In Silico Identification of Novel Targets to Inhibition of Experimental Cerebral Malaria
Source: Pathogens. 2022 Dec 13;11(12):1529. doi: 10.3390/pathogens11121529 (PMC9781199; doi:10.3390/pathogens11121529)
Supplement: Supplementary file 1 [file pathogens-11-01529-s001.zip › pathogens-2082724-supplementary.pdf]

Supplementary Information

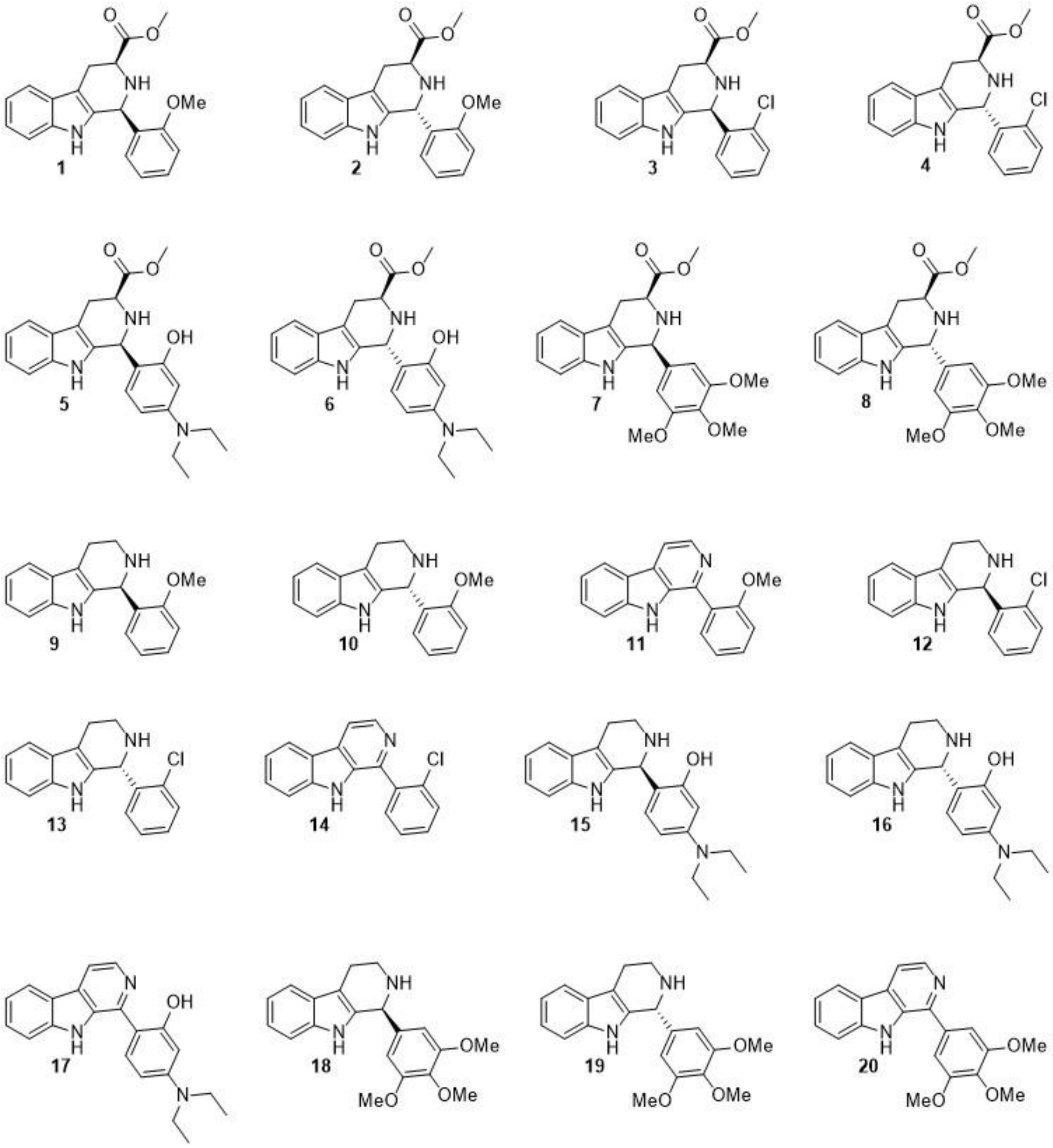

Figure S1. Chemical structure of all compounds possible to be synthesized within the available aldehydes.

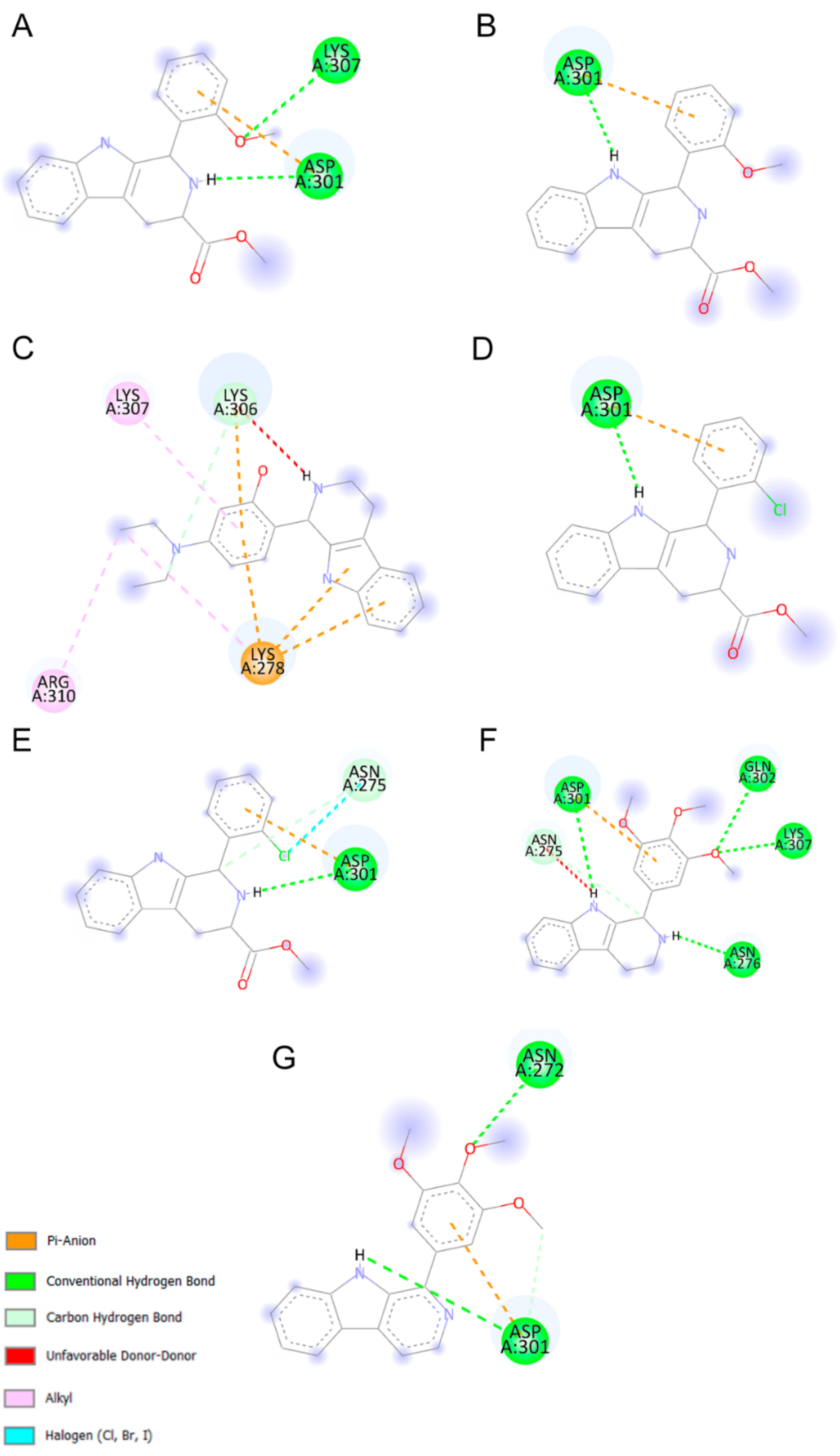

Figure S2. Pharmacophoric map of *in vitro* tested compounds generated by molecular docking against 2OK8; (A) Compound **3**; (B) Compound **4**; (C) Compound **8**; (D) Compound **5**; (E) Compound **6**; (F) Compound **9**; (G) Compound **10**.

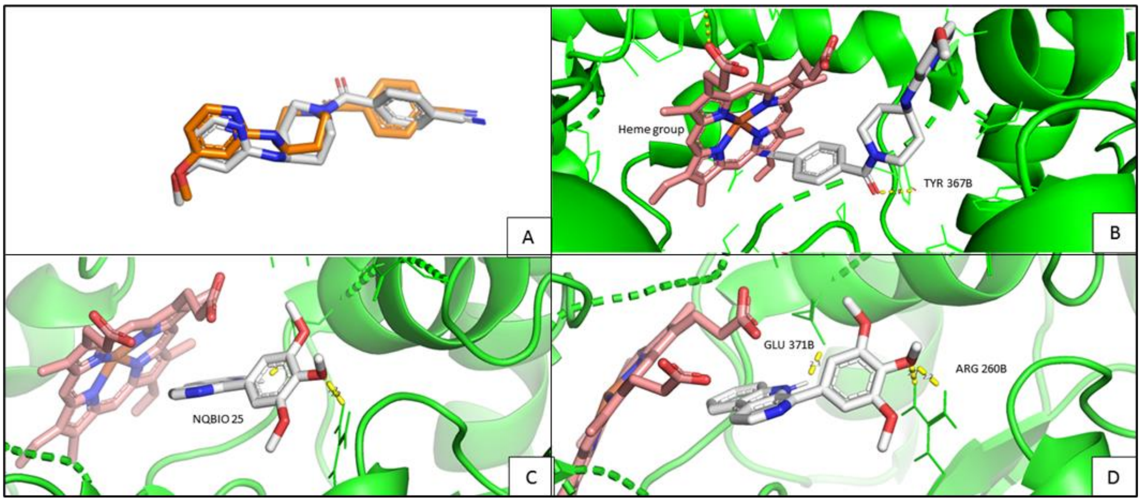

Figure S3. Evaluation of the interaction of compound **10** with the residues of the iNOS active site from Mus musculus. A) Redocking of the crystallographic ligand present in the active site of iNOS. B) Interaction of the crystallographic ligand in the active site of iNOS by hydrogen bonding with TYR 367B residue (Yellow dashed lines). C) Molecular docking of compound **10** in iNOS site. D) Interaction of compound **10** within the active site of iNOS by hydrogen bonding with GLU 371 and ARG 260 residues through hydrogen bonding.
